# Calnexin-Assisted Biogenesis of the Neuronal Glycine Transporter 2 (GlyT2)

**DOI:** 10.1371/journal.pone.0063230

**Published:** 2013-05-01

**Authors:** Esther Arribas-González, Pablo Alonso-Torres, Carmen Aragón, Beatriz López-Corcuera

**Affiliations:** 1 Departamento de Biología Molecular and Centro de Biología Molecular “Severo Ochoa”, Consejo Superior de Investigaciones Científicas, Universidad Autónoma de Madrid, Madrid, Spain; 2 Centro de Investigación Biomédica en Red de Enfermedades Raras, Instituto de Salud Carlos III, Madrid, Spain; 3 IdiPAZ-Hospital Universitario La Paz, Universidad Autónoma de Madrid, Madrid, Spain; University of Pittsburgh, United States of America

## Abstract

The neuronal transporter GlyT2 is a polytopic, 12-transmembrane domain, plasma membrane glycoprotein involved in the removal and recycling of synaptic glycine from inhibitory synapses. Mutations in the human GlyT2 gene (*SLC6A5*) that cause deficient glycine transport or defective GlyT2 trafficking are the second most common cause of hyperekplexia or startle disease. In this study we examined several aspects of GlyT2 biogenesis that involve the endoplasmic reticulum chaperone calnexin (CNX). CNX binds transiently to an intermediate under-glycosylated transporter precursor and facilitates GlyT2 processing. In cells expressing GlyT2, transporter accumulation and transport activity were attenuated by siRNA-mediated CNX knockdown and enhanced by CNX overexpression. GlyT2 binding to CNX was mediated by glycan and polypeptide-based interactions as revealed by pharmacological approaches and the behavior of GlyT2 *N*-glycan-deficient mutants. Moreover, transporter folding appeared to be stabilized by *N*-glycans. Co-expression of CNX and a fully non-glycosylated mutant rescues glycine transport but not mutant surface expression. Hence, CNX discriminates between different conformational states of GlyT2 displaying a lectin-independent chaperone activity. GlyT2 wild-type and mutant transporters were finally degraded in the lysosome. Our findings provide further insight into GlyT2 biogenesis, and a useful framework for the study of newly synthesized GlyT2 transporters bearing hyperekplexia mutations.

## Introduction

Glycine is the major inhibitory neurotransmitter in caudal areas of the vertebrate central nervous system, participating in the motor and sensory information processing involved in movement, vision and audition [1], [2]. Moreover, glycinergic inhibition also modulates nociceptive signaling [3]. The synaptic action of glycine is terminated by its reuptake via specific plasma membrane transporters (GLyTs), which perform high-affinity, active co-transport coupled to the electrochemical gradient of Na^+^ and dependent on Cl^−^ [4]. The neuronal GlyT2 transporter is involved in the removal and recycling of synaptic glycine from inhibitory synapses by supplying substrate to the low-affinity vesicular transporter VIAAT (VGAT) [5]–[8]. Presynaptic glycine taken up by GlyT2 has been demonstrated to be the sole source of releasable transmitter at glycinergic synapses [9], and inactivation of the GlyT2 gene in mice generates a complex postnatal neuromotor phenotype that reproduces the symptoms of human hyperekplexia [6].

Hyperekplexia or startle disease (OMIM 149400) is characterized by neonatal hypertonia and an exaggerated startle response to trivial stimuli [10]. This disorder can lead to brain damage and/or sudden infant death caused by apneic episodes. Genetic analysis of hyperekplexia patients has identified mutations in the human GlyT2 gene (*SLC6A5*) as the second most common cause of the disease, after mutations affecting the glycine receptor and other key proteins in glycinergic synapses [11], [12]. A model of the 3-dimensional structure of GlyT2 was recently developed [13], [14] using as a template the leucine transporter from *Aequifex aeolicus* (LeuT_Aa_), a prokaryotic homologue of the solute carrier 6 (SLC6) family to which GlyT2 belongs [15]. This model provides an explanation for the effects of selected missense mutations on Na^+^- and glycine-binding residues crucial for transport [12], [16]–[19]. The majority of GlyT2 mutations are recessive and cause bi-allelic loss of function due to the absence of the protein in the plasma membrane or the generation of inactive transporters [12]. Only one dominant mutation affecting GlyT2 intracellular trafficking has been described to date (S510R), which is proposed to block the arrival of the transporter to the plasma membrane [16]. However, recently a dominantly inherited mutation that causes complex alterations in transport function and that hinders the proper expression of the transporter at the cell membrane was described [18]. While the trafficking of GlyT2 to and from the plasma membrane along the late secretory pathway has been studied largely [20]–[22], similar analyses of the biogenesis of GlyT2 and its trafficking along the early secretory pathway have not been performed, despite the importance of these processes in the physiology and pathologies of glycinergic neurotransmission.

The synthesis of GlyT2, a polytopic plasma membrane protein, begins with its co-translational translocation to the endoplasmic reticulum (ER) [23]. The biogenesis of these proteins requires interactions with ER chaperones, such as calnexin (CNX), calreticulin (CRT), BiP (GRP78), and oxidoreductases like ERp57 and PDI [24]. CNX is a type I integral membrane protein responsible for the folding and quality control of newly-synthesized glycoproteins [25]. The ER luminal domain of calnexin is responsible for lectin-like activity and interaction with nascent polypeptide chains [26]. Protein-protein interactions may also mediate the interaction with CNX [27], [28]. The second extracellular loop (EL2) of GlyT2 contains 4 asparagines (N345, N355, N360 and N366) that are *N*-glycosylated in the mature protein. The *N*-glycosylation of GlyT2 is partially responsible for its arrival to the plasma membrane, and its asymmetric distribution in polarized cells [29]. In addition, oligomer assembly of neurotransmitter transporters appears to be a prerequisite for export to the plasma membrane and their asymmetric targeting to the neuronal synapse [30].

Here, we confirm a role for CNX in GlyT2 biogenesis, and we describe the kinetics and determinants of the GlyT2-CNX interaction. Our data identify some of the key features of GlyT2 biogenesis in the early secretory pathway. These findings may help to decipher the effects of hyperekplexia mutations on the plasma membrane expression of newly synthesized GlyT2 transporters.

## Materials and Methods

### Cell Growth and Protein Expression

COS7 cells (American Type Culture Collection) were grown at 37°C and 5% CO_2_ in Dulbecco’s modified Eagle’s medium supplemented with 10% fetal bovine serum. Transient expression was achieved using Neofectin™ (MidAtlantic Biolabs), according to the manufacturer’s protocol. Reproducible results were obtained with 50–60% confluent cells on 60 mm or 6 well plates using 5.5 µg and 2.5 µg of total DNA, respectively. Cells were incubated for 48 h at 37°C until used. Transfection efficiency was determined by co-transfecting the cDNAs with the pSV-β-galactosidase plasmid (Promega) and measuring β-galactosidase activity 24 h later after cell solubilization with 25 mM glycylglycine pH 7.8, 0.5% Triton X-100, 1 mM DTT (100 µl/well). After centrifugation (15,000×g) for 2 min, supernatants (15 µl) were transferred to a 96 well plate together with 1 volume of assay buffer (2 mM MgCl_2_, 120 mM NaPO_4_, 80 mM Na_2_HPO_4_, 100 mM β-mercaptoethanol and 1.33 mg/ml O-nitophenyl-β-D-galactopyranoside) and incubated for 20 min at 37°C. Absorbance was measured at 420 nm in an ELISA Dynatech MR5000 and normalized to the protein concentration. Rat brain stem primary neuronal cultures were performed as previously described [29].

### Plasmid Constructs

*N*-glycosylation mutants of rat GlyT2 were inserted into a pCDNA3 vector either as described previously [31] or constructed by site-directed mutagenesis using the QuikChange kit (Stratagene) [32]. Two independent *Escherichia coli* colonies carrying the mutant plasmids were characterized by DNA sequencing and [^3^H]glycine transport activity. We sequenced the complete coding region of each construct to verify that only the desired mutation had been introduced. Mouse cDNA CNX clone (IMAGE number 2582119) was purchased from Source Bioscience Lifesciences.

### Pulse and Chase

Cells cultured to 80–90% confluence in p60 or p100 plates were incubated with methionine-free medium for 1 hour. The cells were then pulse-labeled for 15 min with 0.25 mCi/ml [^35^S]methionine/cysteine (Redivue Promix, Amersham) and chased for varying periods in Dulbecco’s modified Eagle’s medium 10% fetal calf serum containing 1 mM cycloheximide to quickly stop the elongation of nascent polypeptide chains. Labeling was stopped by the addition of ice-cold phosphate-buffered saline (PBS) containing 20 mM freshly prepared N-ethylmaleimide to prevent oxidation of free sulfhydryl groups. Proteins were immunoprecipitated with GlyT2 antibody [33] or sequentially with anti-CNX (Stressgen) and anti-GlyT2 antibodies, as described below. Samples were resolved in SDS-polyacrylamide gels (SDS-PAGE), fixed and treated with Amplify fluorography reagent (Amersham). The gels were dried and exposed for 4–12 days at −70°C, and the protein bands were quantified after densitometry.

### Carbohydrate Modification

Pulse-chased GlyT2 immunoprecipitates were digested with the desired endoglycosidase (PNGase F, New England Biolabs; or Endoglycosidase H or D, Roche) in a small volume of the appropriate buffer, following the manufacturer’s instructions. For tunicamycin treatment, GlyT2-expressing cells were treated with 1–10 µg/ml tunicamycin or the vehicle alone (DMSO) for the time and the temperature indicated in the figure legends, immunoprecipitated with the desired antibodies and resolved by SDS-PAGE.

### Surface Biotinylation

Pulse-chased (or non-labeled) transfected COS7 cells growing in 6 well plates (Nunc) were washed 3 times with complete PBS (PBSc) containing 0.1 mM CaCl_2_ and 1 mM MgCl_2_, and they were incubated for 30 minutes with Sulfo-NHS-Biotin in PBSc (1.0 mg/ml, Pierce) at a temperature that blocks trafficking (4°C). After two 30 min washes at 4°C, 100 mM L-lysine in PBSc was added to block free biotin, cells were rinsed with 150 mM NaCl and 50 mM Tris-HCl (pH 7.4) containing protease inhibitors (0.4 mM PMSF and 4 µM pepstatin) and lysed by end-over-end agitation for 30 minutes at 4°C with 1x lysis buffer (150 mM NaCl, 50 mM Tris-HCl [pH 7.4], 5 mM EDTA, 1% Triton-X, 0.1% SDS, 0.25% deoxycholate sodium, 0.4 mM PMSF and 4 µM pepstatin). A portion of the lysate was saved for total protein determination and the remainder was incubated with streptavidin-agarose for 2 h at room temperature with rotary shaking. After centrifugation, the supernatant was removed (except for an aliquot - not biotinylated), and the agarose beads were washed 3 times with 1x lysis buffer. The bound proteins (biotinylated) were eluted with Laemmli buffer (65 mM Tris, 10% glycerol, 2.3% SDS, 100 mM DTT, 0.01% bromophenol blue) for 10 minutes at 75°C. Samples were analyzed by SDS-PAGE, immunoblotting (Western blot) and densitometry.

### Electrophoresis and Western Blotting

Samples were subjected to SDS-PAGE using a 4% stacking gel and 6% or 7.5% resolving gels. The samples were transferred to nitrocellulose by semi-dry electrotransfer (Life Technologies Inc.: 1.2 mA/cm^2^ for 2 h) and the membranes were then blocked with 5% milk in PBS for 4 h at 25°C. The membranes were probed overnight at 4°C with the desired primary antibody: anti-GLYT2 (1∶1,000), anti-CNX (1∶1,000) or anti-PERK (C33E10, 1∶1,000, Cell Signaling Technology Inc., Danvers, MA). After several washes, the antibodies bound were detected with peroxidase coupled anti-rat or anti-rabbit IgG respectively (1∶6,000), which were visualized by ECL (Amersham Corp.). Subsequently, the antibodies were stripped from the membrane (Thermo Scientific) and it was re-probed with anti-tubulin (1∶3000; Sigma), which was detected with peroxidase-coupled anti-rabbit IgG. The protein bands were quantified by densitometry.

### Inmunoprecipitation Assays

Transfected COS7 cells were washed twice with 20 mM N-ethylmaleimide in PBS and solubilized for 15 min at 4°C in 1 ml of 1% 3-((3-Cholamidopropyl) dimethylammonio)-1-propanesulfonic acid (CHAPS) in HEPES-buffered saline (HBS) buffer (10 mM HEPES-NaOH [pH 7.4], 150 mM NaCl, 1 mM EDTA, 1% CHAPS, 0.4 mM PMSF and 4 µM pepstatin). The CNX-GlyT2 complexes were also maintained in both Triton X-100 and digitonin at the same concentration, although CHAPS displayed the highest solubilization potency. The solubilized material was centrifuged at 10,000×g for 15 min. A portion of the lysate was retained (total protein, input or lysate) and the remainder was incubated with 30 µl of 50% protein A or G cross-linked to sepharose beads in lysis buffer (PAS or PGS: Sigma, St Louis, MO, USA). The mixture was precleared by incubation for 30 min at 4°C with continuous rotation, the samples were centrifuged and the supernatants were incubated for 2 hours at 4°C with 1.5 µg of anti-GlyT2 [33] or anti-CNX antibody (Stressgen Biotechnologies Corp., Victoria, Canada). Subsequently, 30 µl of beads were added and the mixture was incubated for 1 h at 4°C with constant rotation. The beads were washed 3 times with 0.5% CHAPS in ice-cold HBS before adding SDS-PAGE sample buffer to each sample (30 µl). The bound proteins were dissociated from the beads by heating at 75°C for 15 min before SDS-PAGE resolution. For sequential immunoprecipitation, the immunoprecipitated proteins were eluted from the beads by adding 150 µl of 1% SDS in HBS at 75°C for 30 min and centrifuged. The supernatant was diluted with 1.35 ml 1% CHAPS in HBS to decrease the SDS concentration to 0.1% and transferred to a new tube containing 1.5 µg of anti-GlyT2 antibody and incubated overnight at 4°C. The immunocomplexes were bound to beads and eluted as described above. Samples were subsequently subjected to SDS-PAGE.

### siRNA Generation and Transfection

CNX mRNA silencing was achieved by generating and transfecting CNX-specific small d-siRNA into COS7 cells as indicated below. A 300-base pair CNX amplification product (IMAGE number 2582119) flanked by a T7 promoter (RZPD, German Resource Center for Genome Research) was transcribed *in vitro* using the X-tremeGENE siRNA Dicer Kit (Roche) following the manufacturer’s instructions. The resulting annealed dsRNA was subsequently digested with recombinant Dicer enzyme, and the digested RNA was purified and d-siRNA was transfected into COS7 cells using the X-tremeGENE siRNA Transfection Reagent (Roche) with 2.5 µl/0.2 µg d-siRNA. Knockdown efficiency of the CNX-siRNA was assessed in Western blots 48 h post-transfection and it was 85–90%. The siRNA from hypoxanthine phosphoribosyltransferase (HPRT) served as a control.

### Glycine Transport Assays

The day before the assay, the cells transfected with pcDNA3 (control) and/or the cDNAs under study were seeded at 80% confluence in 24 well plates (Nunc). The culture medium was then removed. The cells were washed with PBS (137 mM NaCl, 0.9 mM CaCl_2_, 2.68 mM KCl, 1.47 mM KH_2_PO_4_, 0.49 mM MgCl_2_, 7.37 mM Na_2_HPO_4_ [pH 7.4] and 10 mM glucose) and tempered at 37°C. After a further 10 minute incubation at 37°C with 250 µl of transport medium (2 µl/ml [2-^3^H]-glycine [PerkinElmer Life Sciences], 1.6 TBq/mmol, isotopically diluted to a concentration of 10 µM in PBS), the cells were washed with 750 µl PBS and lysed with 250 µl of 0.2 M NaOH. The protein concentration was determined in aliquots taken from each well by the Bradford method (Biorad) and [2-^3^H]-glycine was measured by liquid scintillation (LKB 1219 Rackbeta). GlyT2 transport was measured as the difference between glycine accumulation in cDNA-transfected cells and that observed in mock-transfected cells. Assays were performed in triplicate or quadruplicate.

### Membrane Isolation

Cells were recovered with PBS 1 mM EDTA and by centrifugation at 1,000 rpm for 5 minutes, resuspended in PBS-EDTA and lysed mechanically by passing the sample 5 times through a needle of 0.5 mm diameter. The lysate was cleared by centrifugation (3,500 rpm for 10 minutes) and the supernatant was collected. The pellet was resuspended in PBS-EDTA and passed 5 times through a needle of 0.3 mm diameter, a step that was repeated twice more. The pellet was discarded and the supernatant collected in the 3 steps above was centrifuged at 18,000 rpm for 45 minutes. The supernatant was discarded and the membrane-enriched pellet was resuspended in a minimal volume of PBS-EDTA. The protein concentration was determined by the Bradford method and adjusted to 0.5 mg/ml.

### Limited Proteolysis

Membranes (25 µl) from an enriched fraction obtained by subcellular fractionation were incubated with 0–100 µg/ml of Papain (Roche) and 0.8 mM DTT in a final volume of 50 µl PBS for 15 minutes at room temperature (22°C). The digestion was stopped by adding 5 mM E-64 (Roche) for 5 minutes on ice and the sample was centrifuged for 90 min at 4°C and 14,000 rpm. The supernatant was discarded and the pellet was resuspended in Laemmli buffer and incubated for 20 minutes at 37°C. Finally, samples were separated by SDS-PAGE and the proteins were visualized by Western blot.

### Data Analysis and Densitometric Quantitation

Protein bands visualized by ECL (Amersham) or by fluorography were quantified in a GS-800 Calibrated Imaging Densitometer using the BioRad Quantity One program with film exposures in the linear range. Non-linear regression fits of experimental transport data were performed using ORIGIN software (Microcal Software, Northampton, MA). The bars represent the S.E.M. of triplicate samples and the representative experiments shown were repeated at least 3 times with comparable results.

## Results

**Figure 1:**
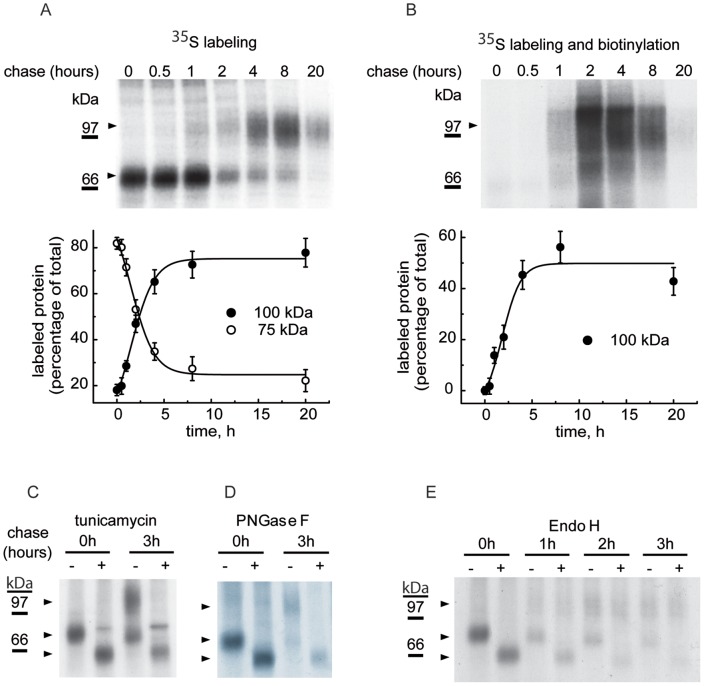
GlyT2 expression after a pulse-chased in culture cells. COS7 cells transfected with GlyT2 cDNA in pCDNA3 were pulse-labeled for 15 min with [^35^S]methionine/cysteine and chased for the times indicated in the conditions given in Material and Methods. The cells were then surface biotinylated, lysed and the protein lysate was either immunoprecipitated with GlyT2 antibody (total transporter, A) or bound to streptavidin-agarose and sequentially immunoprecipitated with GlyT2 antibody (biotinylated fraction, B). Proteins extracted from the beads were resolved in SDS-PAGE. (A) Kinetics of GlyT2 expression. (B) Kinetics of GlyT2 plasma membrane expression in the same cells as in A. Lower panels: (A) densitometry of the 100 kDa and 75 kDa bands in the fluorograms. (B) Biotinylated bands are represented as a percentage of each of the lanes labeled in A. Bars represent the S.E.M. (n = 3). (C) Cells were treated overnight with the vehicle alone (DMSO) or with 10 µg/ml tunicamycin, and then pulse-chased as described above. (D, E) Immunoprecipitates were treated overnight with the vehicle alone (endoglycosidase buffer, -) or with the indicated endoglycosidase (+) in denaturing conditions, and then resolved by SDS-PAGE as described in the Materials and Methods. The transporter proteins (100 kDa, 75 kDa and 60 kDa) are indicated with arrowheads.

To better understand the requirements for the expression of newly synthesized GlyT2 transporters at the plasma membrane, we measured the kinetics of GlyT2 expression in COS7 cells by [^35^S]methionine/cysteine pulse-chase assays, from which GlyT2 was immunoprecipitated with a specific antibody [33]. According to the presence of *N*-glycans attached to GlyT2 at the cell surface [29], electrophoretic separation of the transporter recovered from cultured cells revealed a protein doublet composed of a glycosylated surface and intracellular forms [29], [34]. In our experimental conditions, biosynthesized GlyT2 initially appeared as a 75 kDa metabolic precursor with a half-life of about 1 hour, which then gave rise to a 100 kDa mature transporter (Fig. 1A). The 100 kDa mature protein was present at the plasma membrane and could be labeled with the membrane-impermeant reagent NHS-SS-biotin. The biotinylation of this band increased exponentially during the first 2 hours, reaching a plateau at which it remained for more than 20 hours, suggesting a half-life for this mature protein of over 24 hours (Fig. 1B).

We next employed carbohydrate modification to confirm the glycosylation state of the 75 kDa precursor and to determine its location within the secretory pathway (Fig. 1D, E). Complete enzymatic removal of *N*-linked glycans with PNGase F from both the 75 kDa and the 100 kDa forms after pulse-labeling and subsequent immunoprecipitation yielded a 60-kDa band (Fig. 1D). A protein of the same size was obtained from the 2 protein bands upon treatment of the pulse-chased cells with the *N*-glycosylation blocker tunicamycin, indicating that the 60 kDa form corresponds to the deglycosylated protein core (Fig. 1C). In contrast to the mature transporter, which was resistant to endoglycosidase H (EndoH) digestion, the 75 kDa precursor was EndoH-sensitive at all the time points assayed. This is to be expected for a protein transported via the early secretory pathway. The amount of EndoH-digested protein suggests total EndoH sensitivity, although a general decrease in total GlyT2 labeling, probably due to some contaminant protease activity, was also observed (Fig. 1E). Moreover, the transporter was not sensitive to endoglycosidase D at any time points assayed (data not shown). Taken together, these data indicate that the 75 kDa immature form of GlyT2 is likely to be an underglycosylated form located in the ER or *cis* Golgi [35].

**Figure 2:**
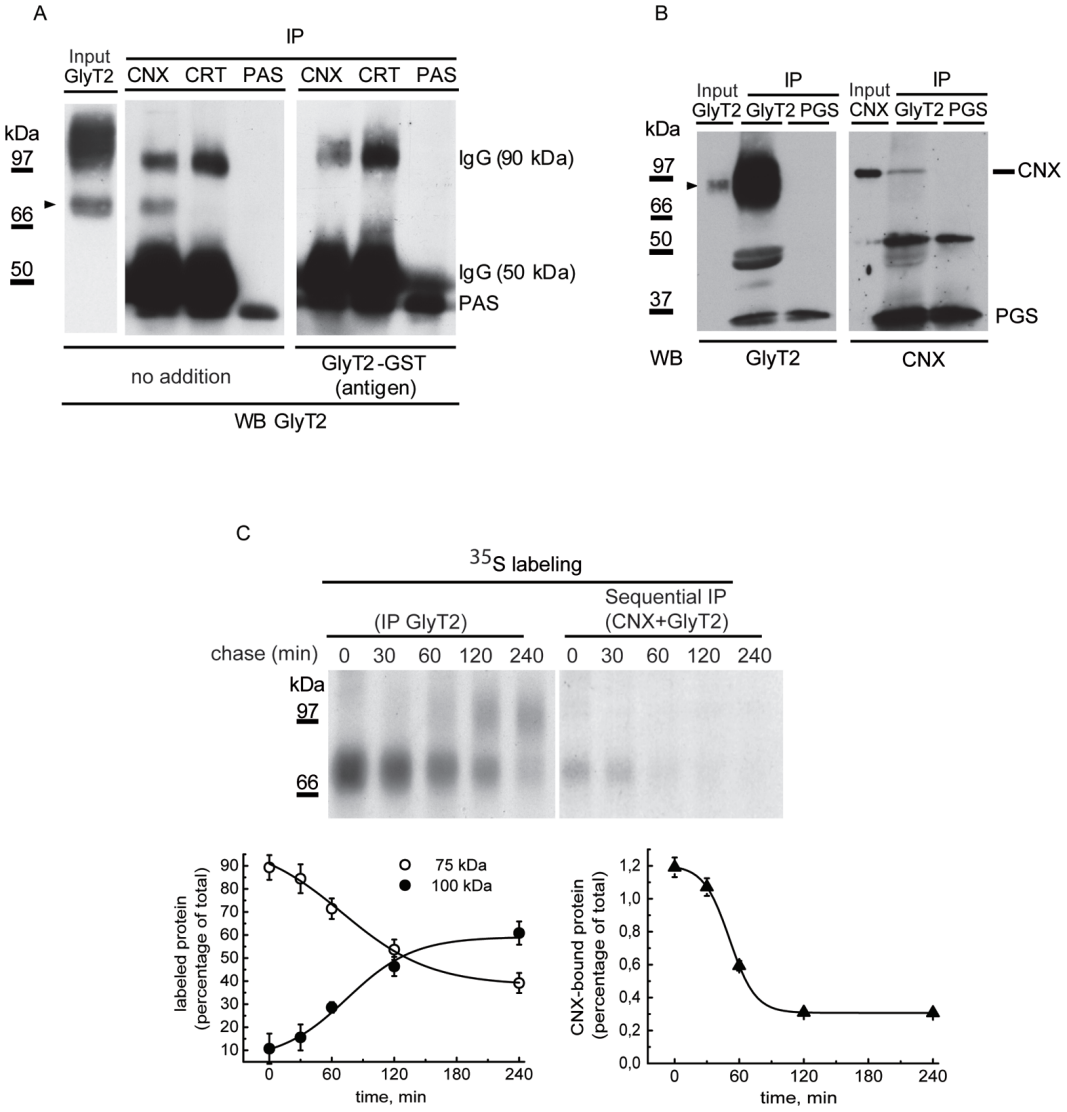
GlyT2 co-immunoprecipitates with CNX. (A) Lysates of COS7 cells expressing GlyT2 were immunoprecipitated with anti-CNX (CNX), anti-calreticulin (CRT) or no antibody (PAS) and then analyzed in Western blots (WB) to detect GlyT2 in the presence or absence (no addition) of 100 µg/ml of the GlyT2 fusion protein used as antigen to generate the rabbit GlyT2 antibody (GlyT2-GST) [30]. The input GlyT2 lane contains 10% of the protein loaded for immunoprecipitation (IP). PAS: protein A sepharose (∼45 kDa). (B) Rat brain stem primary neurons were immunoprecipitated with anti-GlyT2 antibody made in rat [20] and then analyzed in WBs to detect the endogenous immunoprecipitated GlyT2 or CNX by using antibodies made in rabbit. The input lanes contain 5% of the protein loaded for immunoprecipitation (IP). Arrowheads indicate GlyT2. PGS: protein G sepharose (∼17 kDa). (C) COS7 cells expressing GlyT2 were pulse-labeled for 15 min with [^35^S]methionine/cysteine, chased for the times indicated and subjected to sequential immunoprecipitation with CNX and GlyT2 antibodies, as described in the Materials and Methods. Lower panel: densitometry of the fluorograms.

**Figure 3:**
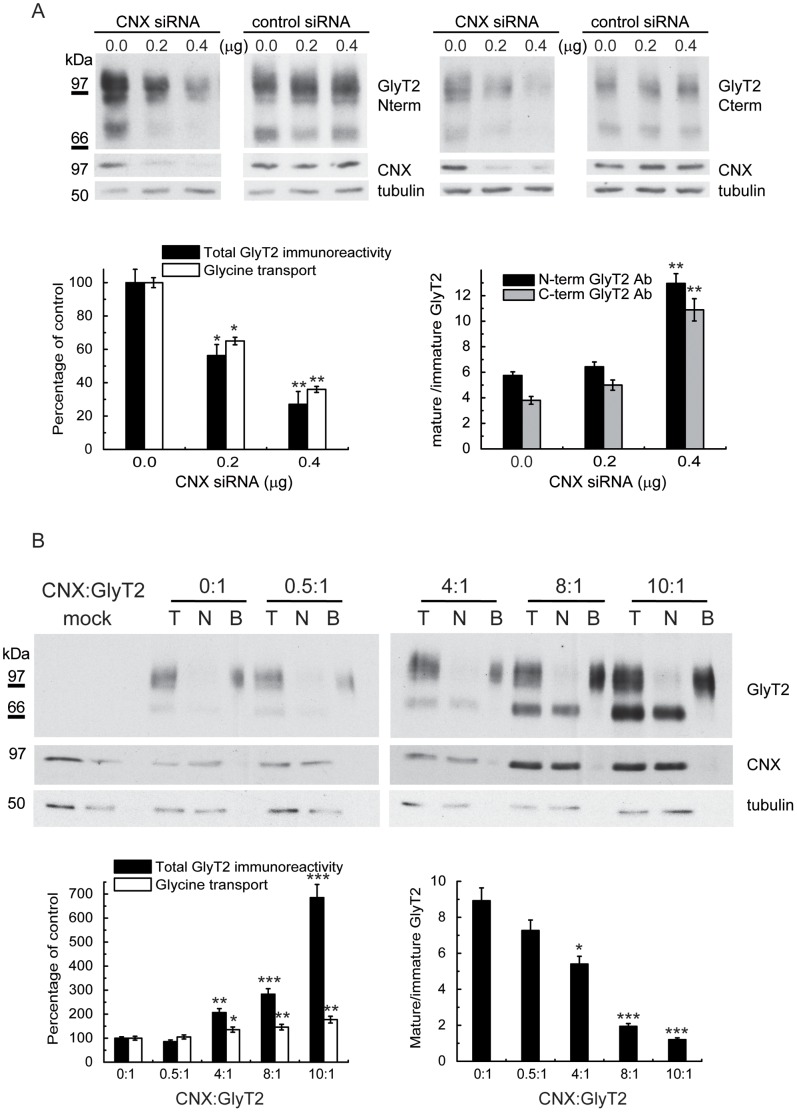
Expression of GlyT2 following CNX knockdown/overexpression. (A) COS7 cells were co-transfected with 0.5 µg of GlyT2 cDNA in pCDNA3 and the indicated amount of control (HPRT) or CNX siRNA. At 48 h post-transfection, the cells were analyzed in Western blots (upper panel) or assayed for glycine transport (open bars, left graph). The specific CNX d-siRNA reduced CNX protein levels by 62% (0.2 µg) and 85% (0.4 µg), respectively, as compared with endogenous levels. Control d-siRNA increased total GlyT2 levels by 10% and 15%, respectively. Right graph: ratio of mature (100 kDa) to immature (75 kDa) band at the different amounts of CNX siRNA transfected. The bands were detected with GlyT2 antibodies against N- or C-terminal epitopes (Fig. S1). (B) COS7 cells were co-transfected with 0.5 µg of GlyT2 cDNA together with a CNX cDNA at the indicated mass ratio (CNX:GlyT2). At 48 h post-transfection the cells were biotinylated (T = total transporter; N = non-biotinylated transporter; B = biotinylated transporter, 3-fold the protein amount in T or N) or glycine transport was assayed (open bars, left histogram). Solid bars in the left histogram represent total GlyT2 normalized to tubulin immunoreactivity. Verification of CNX overexpression by densitometry revealed the following increases at increasing mass ratios: 0∶1, 1-fold (endogenous CNX); 0.5∶1, 1.8-fold; 4∶1, 2.4-fold; 8∶1, 9.3-fold; 10∶1, 12.9-fold. Right graph: the ratio of the mature (100 kDa) to immature (75 kDa) protein decreased with the amount of CNX expressed. Bars represent the S.E.M (n = 6). *p<0.05, **p<0.01, ***p<0.001 with respect to control (Student’s t-test).

The ER is the site at which quality control of the glycoproteins synthesized takes place, with the assistance of molecular chaperones such as calnexin (CNX) [36]. The 75 kDa GlyT2 precursor co-immunoprecipitated with an antibody against CNX and its immunodetection in Western blots was prevented by antigen preadsorption with an excess of the GlyT2 fusion protein (GlyT2-GST) previously used to generate the GlyT2 antibody [33]. These observations confirmed the immunoprecipitated 75 kDa band to be the transporter precursor (Fig. 2A). The co-immunoprecipitation of GlyT2 and CNX also occurred in primary neurons in which GlyT2 appeared as a lower molecular weight band that overlapped with that of the precursor (Fig. 2B). However, GlyT2 did not immunoprecipitate with calreticulin in any of the conditions assayed (Fig. 2A). We next measured the kinetics of the GlyT2-CNX interaction by performing sequential immunoprecipitation of GlyT2-expressing pulse-chased COS7 cells using CNX and GlyT2 antibodies (Fig. 2C). The 75 kDa precursor transiently associated with CNX to form a complex with a half-life of about 60 min, which agreed with the onset of the expression of the 100 kDa mature transporter, suggesting that CNX facilitates GlyT2 biogenesis. The CNX-bound GlyT2 was deglycosylated by PNGaseF, it was sensitive to Endo H and was Endo D resistant, as expected of the GlyT2 precursor present in the ER or *cis* Golgi (not shown). In agreement with a facilitatory role of CNX in GlyT2 biogenesis, total expression of the transporter was sensitive to the expression of the chaperone (Fig. 3). Knockdown of CNX in COS7 cells using a specific RNAi reduced the total transporter expression (as evident in Western blots) and decreased glycine transport activity in a dose-dependent manner (Fig. 3A), indicating that the low levels of remaining CNX limit GlyT2 synthesis. Consequently, accumulation of the mature form of the transporter was observed, resulting in a progressive increase in the mature (100 kDa)/immature (75 kDa) ratio in steady state conditions. This increase is not due to a differential recognition of the immature protein by the used GlyT2 antibody, since the same result was obtained with an antibody against the C-terminus of GlyT2 (Fig. 3A and Fig. S1). Conversely, with an optimized co-transfection protocol in COS7 cells we found that CNX overexpression dramatically increased total GlyT2 expression and glycine transport. Furthermore, in agreement with the increased number of binding sites for the GlyT2 precursor following CNX overexpression, we also detected an increased proportion of immature transporter (Fig. 3B). Together, these results confirm that GlyT2 biosynthesis is assisted by CNX.

**Figure 4:**
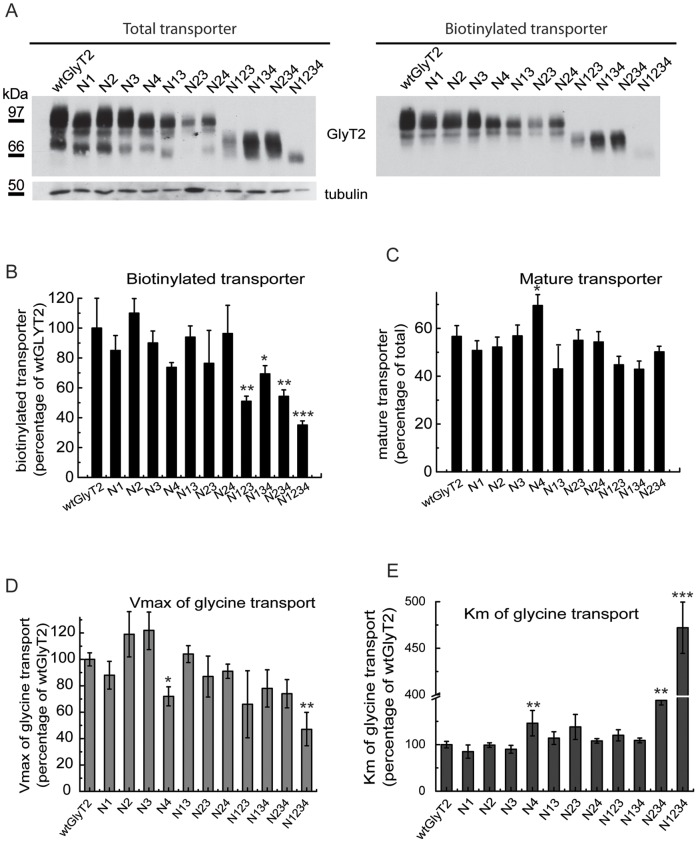
Characterization of GlyT2 *N*-glycan site mutants. (A) COS7 cells expressing the indicated GlyT2 *N*-glycosylation mutants were biotinylated and processed as described in the Materials and Methods. (A) Lysates (6 µg, total transporter, left) or biotinylated protein (18 µg, biotinylated transporter, right) were resolved by SDS-PAGE and analyzed in Western blots to detect GlyT2. Tubulin immunoreactivity was used as a loading control. Immunoblots were quantified by densitometry. (B) Densitometry representing the proportion of mutant biotinylated transporter as a percentage of the biotinylated fraction of wild-type GlyT2. (C) Densitometry of the Western blots expressing the fraction of mature transporter (upper band) as a percentage of the total transporter for each mutant. (D,E) COS7 cells expressing the indicated GlyT2 *N*-glycosylation mutants were assayed for glycine transport (as described in the Materials and Methods) in the presence of increasing concentrations of glycine from 1 to 1000 µM. Kinetic data were fitted to hyperbolae and V_max_ and K_m_ parameters were calculated from the best fit. For wild-type GlyT2, V_max_ = 17±3.8 nmol gly/mg prot/4 min and K_m_ = 171±20 µM. Bars represent the S.E.M (n = 3). *p<0.05, **p<0.01, ***p<0.001 with respect to wild-type GlyT2 (ANOVA with Tukey’s post-hoc test).

We previously demonstrated that GlyT2 contains 4 *N*-glycan chains attached to asparagines 345, 355, 360 and 366 in the mature protein [29]. As a monovalent lectin, CNX has affinity for monoglucosylated intermediaries but it can also associate via protein-protein interactions [27], [28]. We constructed various mutants with deficiencies in single or multiple *N*-glycan acceptor sites by substituting the asparagines 345, 355, 360 or 366 with aspartates [29]. The single (N1, N2, N3 and N4) and double (N13, N23, N24) mutants maintained a wild-type-like time course of expression in [^35^S] pulse-chased COS7 cells (data not shown). The onset of the appearance of the glycosylated protein in the singly glycosylated triple mutants (N123, N134, N234) could not be accurately determined in pulse-chased cells due to the small size of the mature transporter close to the [^35^S]labeled immature precursor. However, we observed a decrease in the amount of biotinylated plasma membrane transporter (Fig. 4A–C) that paralleled transporter activity (Fig. 4D,E). The Vmax of glycine transport diminished progressively and the Km increased markedly, reaching levels in the fully deglycosylated mutant (N1234) that were 5-fold higher than those of the wild-type GlyT2. This increase in Km was also observed when *N*-glycosylation mutants were generated by replacing the asparagines with glutamines rather than aspartates (data not shown), indicating that the sugar rather than the charge removal caused the increase in Km. One important exception in this series of mutants was the N4 mutant (N366). Although its time course of expression was comparable with that of the wild-type, this mutant exhibited slightly slower mature protein synthesis (Fig. S2), reduced levels of total biotinylated surface transporter (Fig. 4A,B), an increased proportion of glycosylated versus total transporter (Fig. 4C), a lower Vmax (Fig. 4D) and a higher Km than the other related mutants (Fig. 4E). In addition, the glycosylated N4 band was of a lower apparent weight than N2 and N3 (Fig. 4A), suggesting that either the *N*-glycans attached to N366 have a greater molecular mass or that their removal affects GlyT2 folding. We hypothesized that some of the features displayed by the N4 mutant were sustained by reduced binding to CNX (see below). We therefore further investigated the factors that influence GlyT2 binding to the chaperone.

**Figure 5:**
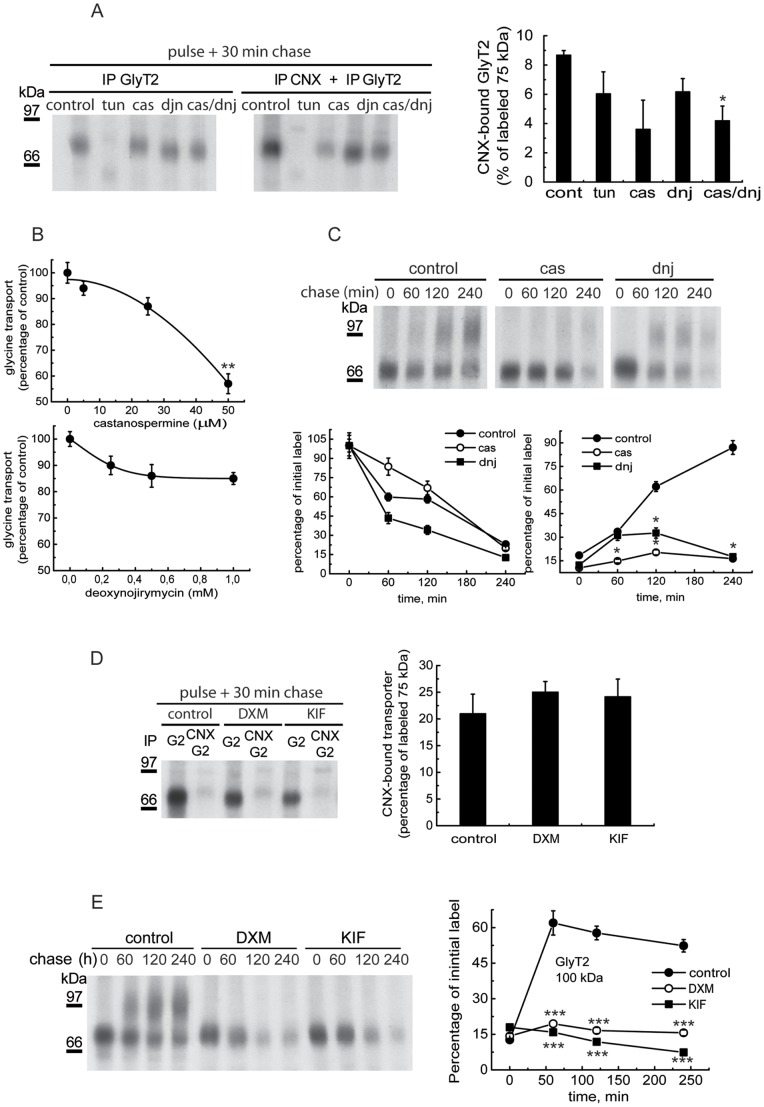
Effects of glucosidase and mannosidase inhibitors on CNX binding and GlyT2 maturation. (A) COS7 cells expressing GlyT2 were treated for 2 h with the vehicle alone (DMSO), 1 µg/ml tunicamycin, 1 mM castanospermine (cas), 1 mM deoxynojirimycin (dnj) or 1 mM castanospermine plus 1 mM deoxynojirimycin (cas/dnj), and then pulse-labeled for 15 min with [^35^S]methionine/cysteine and chased for 30 min. The cell lysates were then immunoprecipitated with a GlyT2 antibody or subjected to sequential immunoprecipitation with CNX and GlyT2 antibodies (as described in the Materials and Methods) and resolved by SDS-PAGE. Right panel: densitometry of the fluorograms (n = 3) showing the percentage of total GlyT2 precursor bound to CNX in each condition (± S.E.M). (B) COS7 cells expressing GlyT2 were treated for 2 h with the vehicle alone (water), 1–50 µM castanospermine (upper graph) or 0.2–1 mM deoxynojirimycin (lower graph) and then glycine transport was assayed in the cells. (C) COS7 cells expressing GlyT2 were treated for 2 h with the vehicle alone (control), 1 mM castanospermine or 2 mM deoxynojirimycin and then pulse-labeled for 15 min with [^35^S]methionine/cysteine, chased for the times indicated. The cell lysates were immunoprecipitated with GlyT2 antibody and analyzed by SDS-PAGE. Lower graphs: densitometry. (D,E) The experimental conditions applied were as described in A and C, except the cells were treated with 1 mM deoxymannojirimycin (DXM) or 10 µM kifunensin (KIF). *p<0.05, **p<0.01, ***p<0.001 with respect to controls (Student’s t-test).

**Figure 6:**
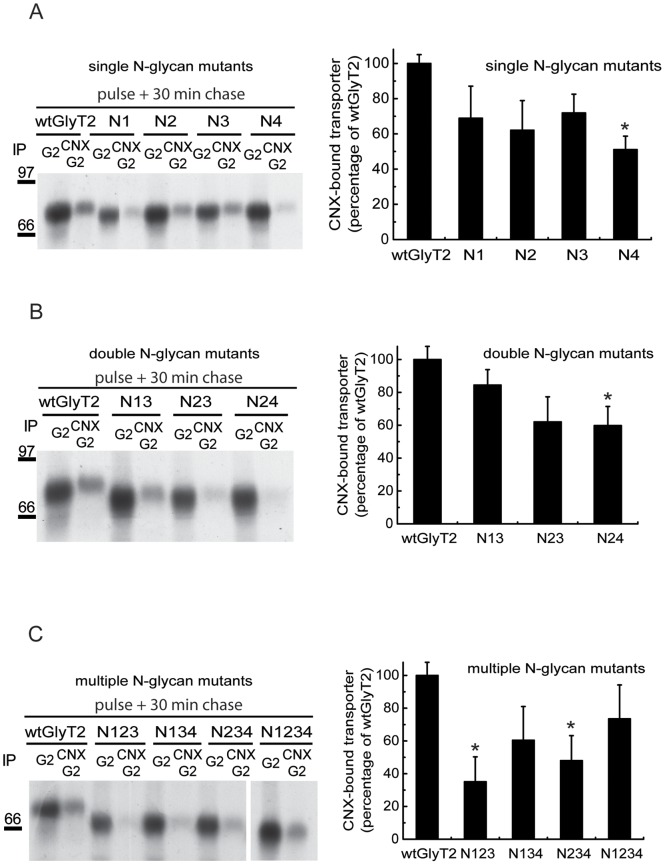
Co-immunoprecipitation of GlyT2 *N*-glycan mutants and CNX. COS7 cells expressing the indicated single (A), double (B) or multiple (C) *N*-glycan mutants were pulse-labeled for 15 min with [^35^S]methionine/cysteine and chased for 30 min. The cell lysates were immunoprecipitated with GlyT2 antibody or subjected to sequential immunoprecipitation with CNX and GlyT2 antibodies (as described in the Materials and Methods) and analyzed by SDS-PAGE. Right panels: densitometric analysis of the fluorograms (n = 3) showing the percentage of total GlyT2 precursor bound to CNX in each condition (± S.E.M). *p<0.05 with respect to wild-type GlyT2 (ANOVA with Tukey’s post-hoc test).

Glucosidases I and II (GI and GII) give rise to monoglucosylated CNX substrates [37]. Treatment of GlyT2-expressing COS cells with the glucosidase inhibitors castanospermine and deoxynojirimycin resulted in a partial but consistent reduction in the levels of CNX-bound transporter recovered by sequential immunoprecipitations (Fig. 5A). CNX binding was also sensitive to low concentrations of tunicamycin, an inhibitor of *N*-glycosylation. Moreover, glucosidase inhibition impaired glycine transport activity (Fig. 5B) as a consequence of decreased transporter maturation (Fig. 5C). By contrast, mannosidase inhibition did not reduce the amount of CNX-bound GlyT2, although it fully prevented the generation of the mature 100 kDa transporter (Fig. 5D,E). The latter observation was expected as mannose trimming is not required for the generation of CNX substrates but it is necessary for the further processing of glycans in the Golgi [28]. The increased specificity and membrane permeability of mannosidase versus glucosidase inhibitors may explain the greater efficiency of the former in preventing the expression of the mature transporter [38]. The loss of sugar following mutagenesis also hampered the interaction with CNX measured after a 15-min pulse and a 30-min chase. However, these experiments revealed no correlation between glycan removal and CNX binding. Indeed, depending on the single or multiple *N*-glycan-deficient mutant chosen, 60–80% of the CNX binding observed in the wild-type was obtained (Fig. 6A,B). Moreover, the removal of 3 or 4 *N*-glycosylation sites did not further impair CNX interaction. In fact, a considerable amount of *N*-glycosylation-deficient GlyT2 mutant was bound to CNX and recovered by sequential immunoprecipitation (Fig. 6C), suggesting that the association of GlyT2 with CNX is mediated by interactions involving both sugars and polypeptides. Accordingly, the reduced CNX binding exhibited by the N4 mutant was possibly due to the involvement of N366 in CNX binding or to an alteration in the conformation of GlyT2 following *N*-glycan removal.

**Figure 7:**
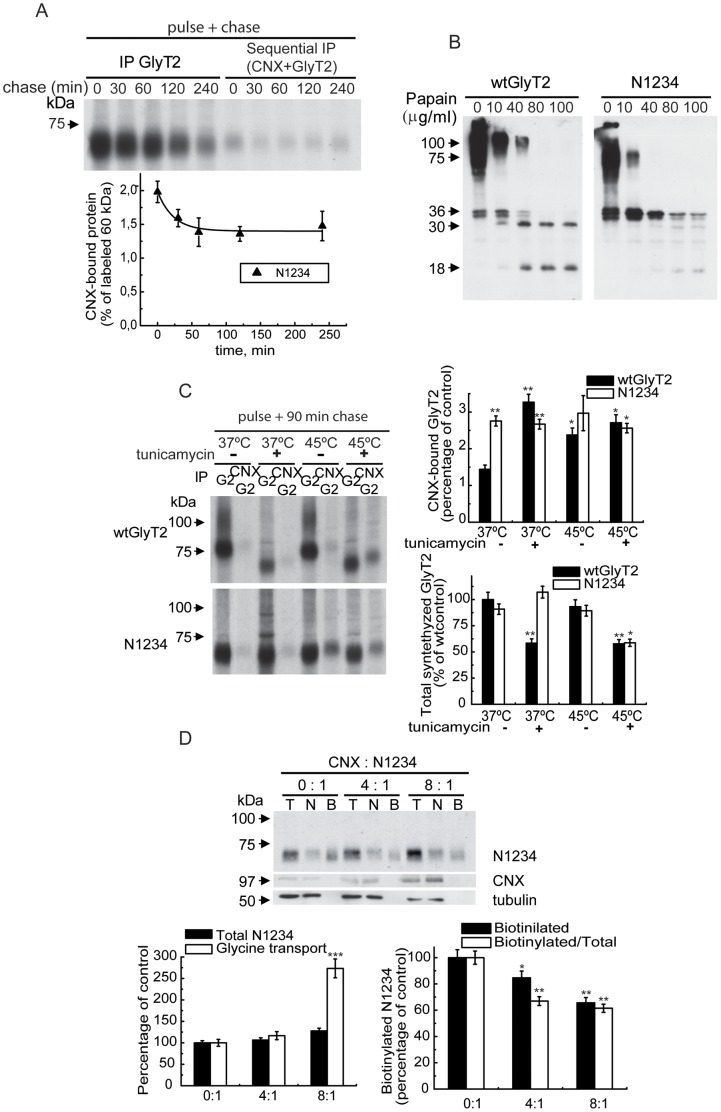
CNX-binding and folding properties of the *N*-glycan-deficient GlyT2 mutant. (A) COS7 cells expressing the N1234 *N*-glycan-deficient mutant were pulse-labeled for 15 min with [^35^S]methionine/cysteine and chased for the indicated time. The cell lysates were immunoprecipitated with GlyT2 antibody or subjected to sequential immunoprecipitations with CNX and GlyT2 antibodies as described in the Materials and Methods and resolved by SDS-PAGE. Lower graph: densitometry of the fluorograms (n = 2) showing the percentage of N1234 bound to CNX in each condition (± S.E.M). (B) The membrane enriched fraction from COS7 cells expressing GlyT2 or the N1234 *N*-glycan-deficient mutant (12.5 µg) was digested for 15 min at 22°C with the concentrations of papain indicated as described in the Materials and Methods and analyzed in Western blots probed with antibodies against the GlyT2 N-terminus. (C) COS7 cells expressing wild-type GlyT2 or the N1234 mutant were treated with the vehicle alone (-) or 10 µg/ml tunicamycin (+) for 3 h at 37°C, pulse-labeled for 15 min with [^35^S]methionine/cysteine and then chased for 90 min at the temperature indicated. The cell lysates were immunoprecipitated with GlyT2 antibody or subjected to sequential immunoprecipitation with CNX and GlyT2 antibodies as described in the Materials and Methods and resolved by SDS-PAGE. Right panels: densitometry of the fluorograms (n = 3) showing the percentage of total GlyT2 precursor bound to CNX in each condition (up) and the total synthesized transporter (down). Bars represent the S.E.M. (n = 3). *p<0.05, **p<0.01 with respect to wild-type control (Student’s t-test). (D) The experimental conditions applied were as described in Figure 3B except that cells expressed the N1234 mutant and CNX overexpression was achieved by co-expressing CNX and N1234 at ratios of 0∶1, 4∶1 and 8∶1. *p<0.05, **p<0.01, ***p<0.001 with respect to the wild-type control (Student’s t-test).

The behavior of the N1234 mutant, which lacks *N*-glycan acceptor sites, provided further evidence of the involvement of protein-protein interactions in GlyT2 binding to CNX (Figs. 4 and 7). In contrast to wtGlyT2, which associated transiently with CNX (Fig. 2C), the mutant engaged in a more persistent interaction with the chaperone, as revealed by sequential immunoprecipitation of pulse-chased cells with CNX and GlyT2 antibodies (Fig. 7A). This finding was consistent with the reduced surface expression and V_max_ of glycine transport shown by this mutant (Fig. 4A,B,D), and suggests that the unglycosylated transporter does not easily pass CNX quality control, an ER check point for newly synthesized glycoproteins [39]. This longer lasting binding may reflect an alteration to the 3-dimensional structure of the mutant that is detected by CNX. Indeed, the mutant displayed increased proteolytic sensitivity in a limited proteolysis experiment using the wild-type and N1234 mutant and increasing concentrations of papain, such that low molecular weight proteolytic fragments (36, 34, 30 and 18 kDa) that could be detected with an antibody against the N-terminal region of GlyT2 were produced at different protease concentrations (Fig. 7B). This may be indicative of misfolding in the mutant, although glycan-mediated protection from proteolysis is also possible.

As well as inhibiting *N*-glycosylation, tunicamycin may also trigger the unfolded protein response (UPR) and therefore act as a misfolding agent when used at high concentrations or long incubation times [40]. Hence, we performed pulse-chase experiments in the presence of this inhibitor with longer chase times (90 min) and at different temperatures (Fig. 7C). In the presence of high concentrations of tunicamycin, the association of wild-type GlyT2 to CNX was enhanced at 37°C and to a much lesser extent at 45°C, suggesting that this association is enhanced by tunicamycin-induced unfolding. The effect of tunicamycin was less evident at 45°C as both tunicamycin and high temperature treatment trigger general unfolding (Fig. 7C, upper histogram). By contrast, the N1234 mutant displayed greater CNX binding in all the conditions assayed, and its association to CNX was insensitive to tunicamycin or the increase in temperature. The same pattern was observed in assays using thapsigargin, another inducer of UPR (data not shown). Moreover, the total amount of wild-type transporter synthesized at 37°C was reduced in the presence of tunicamycin, while that of the mutant was tunicamycin-insensitive. As expected, there was a general reduction in total protein synthesis at 45°C (Fig. 7C, lower histogram). These results strongly suggest that the 3-dimensional structure of the N1234 mutant lacking sugar chains is altered, consistent with its proteolytic pattern and with the dramatic increase in the K_m_ for glycine transport shown by this mutant (Fig. 7B and 4E).

To determine whether CNX can act as a *bona-fide* chaperone independently of its lectin activity, we investigated the effect of CNX overexpression in cells expressing the N1234 mutant using the experimental conditions shown in Fig. 3B for wtGlyT2 (Fig. 7D). In agreement with the effect of CNX on the wild-type GlyT2, total expression of the mutant and its glycine transport were enhanced by CNX overexpression, albeit to a lesser extent (Fig. 7D, left histogram). Conversely, surface expression of the mutant transporter was reduced by overexpressing the chaperone (Fig. 7D, right histogram), suggesting that it was more efficiently folded although less of the mutant transporter reached the plasma membrane. These findings suggest that CNX can discriminate between different conformational states in a glycan-independent manner and select the most functionally competent structure required.

**Figure 8:**
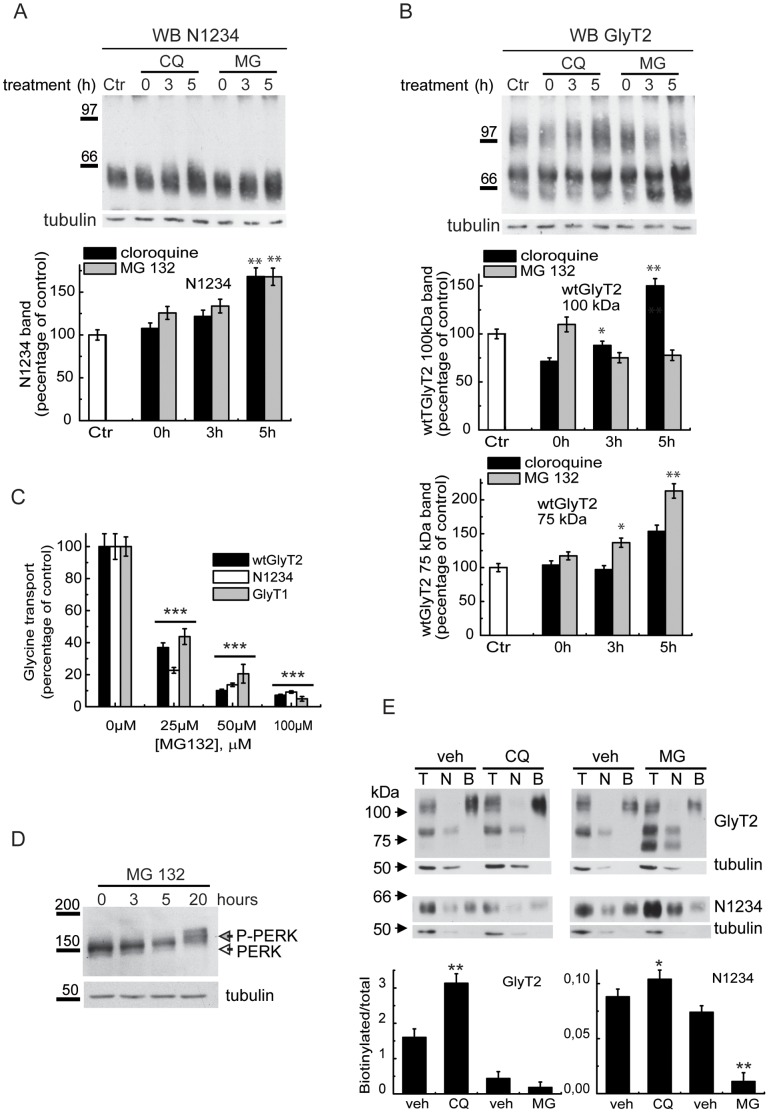
Degradation of GlyT2. (A,B) COS7 cells expressing the N1234 mutant or wild-type GlyT2 were treated with 0.1 mM chloroquine (CQ), 50 µM MG132 (MG) or the corresponding vehicles (water and DMSO, respectively) for the times indicated, resolved by SDS-PAGE and GlyT2 was analyzed in Western blots. Densitometry (n = 2–4) is shown in the histograms in A and B. *p<0.05, **p<0.01 with respect to control at the corresponding time points (Student’s t-test). (C) COS7 cells expressing wtGlyT2, N1234 or a control protein (GlyT1) were treated with vehicle or MG132 for 16 h at the concentrations indicated and glycine transport was then assayed in the cells. Bars represent the S.E.M. (n = 3). ***p<0.001 with respect to control (Student’s t-test). (D) COS cells were treated with vehicle or 5 µM MG132 for the times indicated and then resolved in SDS-PAGE and PERK was analyzed in Western blots. Tubulin immunoreactivity was used as a loading control in A, B and D. (E) COS7 cells expressing the N1234 mutant or wild-type GlyT2 were treated for 5 hours with 0.1 mM chloroquine (CQ), 50 µM MG132 (MG) or the corresponding vehicle solutions (water and DMSO, respectively), biotinylated and GlyT2 was analyzed in Western blots. *p<0.05 with respect to wild-type control (Student’s t-test).

The long-term binding of the glycosylation-deficient mutant to CNX suggests that it fails to progress past a quality control check point. This may result in the transporter being sent for proteasomal degradation, the ultimate fate of deficiently-folded membrane proteins [41]–[43], or it may be resolved by correct folding and subsequent progression through the secretory pathway. Treatment of N1234-expressing COS7 cells with the lysosome inhibitor chloroquine, or with high concentrations of the proteasome inhibitors MG132 and lactacystin (data not shown), resulted in comparable increases in the amount of mutant transporter (Fig. 8A). This may indicate that the mutant uses both lysosomal and proteasomal pathways for degradation. However, the same treatments applied to wild-type cells expressing GlyT2 produced opposite effects: while treatment with the lysosome inhibitor chloroquine augmented the mature (100 kDa) transporter, proteasome inhibition reduced the amount of mature transporter (Fig. 8B). In addition, a band of ∼60 kDa visibly augmented in immunoprecipitates from cells treated with proteasome inhibitor. The apparent molecular weight of this band coincided with that of the deglycosylated protein core (Fig. 1), suggesting that it represents an early arrested form of the transporter. This raises the possibility that proteasome inhibition in our experimental conditions induced ER stress and affected general ER protein folding.

To further investigate this possibility we studied the effect of increasing MG132 concentrations on glycine transport by wtGlyT2, the N1234 mutant and a control protein (GlyT1). All 3 transporters were similarly sensitive, suggesting that off-target secondary effects of proteasome inhibition may produce a general folding defect (Fig. 8C). Indeed, the phosphorylated form of protein kinase-like endoplasmic reticulum kinase (PERK), a well-established ER stress marker that is activated during UPR and that attenuates mRNA translation by phosphorylating eIF2α [44], was already visible when the cells were treated for 3 h with a low concentration of MG132 (5 µM; Fig. 8D). Thus, a clearly involved degradation pathway for both wtGlyT2 and the N1234 mutant appears to be the lysosome, as both seem to accumulate in the presence of lysosome inhibitors (Fig. 8E). However, this effect was more evident for the wild-type especially over longer chase times (Fig. S3), leading us to consider a contribution of the proteasome in the degradation of the mutant.

## Discussion

According to the validated LeuTAa homology model for the SLC6 family of sodium- and chloride-dependent transporters, the plasma membrane GlyT2 has 12 transmembrane domains (TM) oriented such that TMs 1–5 are positioned in an antiparallel conformation relative to TMs 6–10 [13]–[15]. While resolving the crystal structure of the prokaryote transporter has furthered our understanding of the underlying protein structure significantly, the biosynthesis of polytopic plasma membrane proteins like GlyT2 and the role of the *N*-glycans attached to its EL2 remain poorly understood. Nascent GlyT2 is translocated to the lumen of the ER and oligosaccharide moieties are co-translationally attached to 4 EL2 asparagines in *N*-glycosylation consensus sequences (N345, N355, N360 and N366) [29]. CNX/CRT retains glycoproteins in the ER by re-binding to the lectin site until a folded conformation of the substrate is achieved. Properly folded glycoproteins are incorporated into transport vesicles and exported to the Golgi, while terminally misfolded glycoproteins that persist in CNX/CRT cycles are targeted for degradation by the ER-associated degradation (ERAD) pathway [28], [36].

Our electrophoretic analysis of GlyT2-expressing cells revealed a 75 kDa precursor that disappeared as the chase times following [^35^S]-labeling increase. This protein represents the ER precursor that has received the initial 14-sugar chain from dolichol and carbohydrate removal with PNGase F or tunicamycin yielded a 60 kDa protein core. This is smaller in size than the protein predicted by summing the molecular weights of the individual amino acid residues (78.9 kD), although packing of the protein fraction may account for this weight difference, as previously described for other related transporters [45]. At longer chase times, the fully glycosylated transporter appeared as a 100 kDa diffuse band, which may contain a mixture of glycosylated species in different states of trimming. We investigated several aspects of GlyT2 biogenesis in which CNX plays a facilitatory role. By performing sequential immunoprecipitation with CNX followed by GlyT2 antibodies, we captured the pulse-labeled 75 kDa CNX-bound precursor in the ER. The CNX-bound precursor reached maximal levels immediately after the pulse, before the appearance of the mature 100 kDa transporter, and then decayed to basal levels. As expected for CNX-assisted biogenesis, GlyT2 was very sensitive to CNX concentrations, and siRNA-mediated CNX knockdown in GlyT2-expressing cells reduced membrane expression and transport function by limiting the amount of the available precursor. These results are in good agreement with the phenotype of CNX knockout mice, which die after 4 weeks due to the inability of CRT to fully substitute CNX activity [46]. We detected no GlyT2 binding to CRT in any of the conditions assayed, which included a range of different detergents and chase times. On the other hand, optimizing the co-transfection protocol to use low levels of transfected cDNA and prevent competition for the cell translation machinery, we demonstrated that the access of GlyT2 to the membrane was facilitated dramatically following CNX overexpression. CNX made possible the maturation of a greater proportion of immature transporter and increased levels of transporter at the cell surface and thus transport activity. These results are in good agreement with earlier studies of other SLC6 transporters [47], and strongly support the role of CNX as a chaperone in GlyT2 biogenesis.

GlyT2 binding to CNX is mediated by glycans and protein-protein interactions, as revealed by disruption of transporter binding with pharmacological treatments and through the behavior of GlyT2 mutants lacking *N*-glycan acceptor sites. GlyT2 binding to CNX is sensitive to GI and GII inhibitors. Deoxinojirimycin is a more potent GII inhibitor than castanospermine, yet the latter inhibits the activity of both GI and GII [48], [49]. The differential effect of these inhibitors on GlyT2 biosynthesis may be linked to the crucial role of GI in rapid glucose trimming after glycan addition or the modulation of GII activity by neighboring *N*-glycans, which may attenuate the inhibition of GlyT2 [36], [37]. However, the cell permeability of both these compounds is poor, and although IC_50_ values in the low micromolar range have been measured in cell-free systems, these values increase by several orders of magnitude in cell cultures [38]. This may explain why these glucosidase inhibitors exhibited variable effects and why high concentrations were required to prevent GlyT2 maturation. However, even using concentrations in the high mM range, we were unable to fully prevent GlyT2 binding to CNX, indicating that CNX binding requires polypeptide- as well as lectin-based interactions [36], [39].

The binding of GlyT2 to CNX was also sensitive to the removal of single or multiple *N*-glycan sites, although it increased most when all *N*-glycan binding sites were removed. This is consistent with the hypothesis that initial binding to calnexin requires monoglucosylated *N*-linked oligosaccharides until the mature protein conformation is achieved, although the retention of bound proteins is not mediated by the glycans but by polypeptide motifs [50], [51]. Furthermore, for GII to generate the monoglucosylated oligosaccharide in mammalian cells, at least 2 oligosaccharide chains on the substrate glycoprotein are required [37]. The removal of multiple glycosylation sites may alter the protein conformation and unmask unfolded peptide regions that are detected by CNX, thereby increasing binding. This may be due to a chaperone-independent pro-folding effect of *N*-glycosylation, as reported for the cystic fibrosis transmembrane regulator [52]. We previously demonstrated that PNGase F treatment of the purified and reconstituted GlyT2 impairs glycine transport, probably due to the anomalous conformation of the deglycosylated protein [29]. In the present study we show that the GlyT2 N1234 mutant, which lacks all 4 *N*-glycan acceptor sites, exhibits anomalous folding as revealed by differential papain and pronase (not shown) proteolysis patterns. Accordingly, this mutant displays deficient glycine transport in functional assays, as revealed by a low Vmax and high Km. Moreover, membrane expression of the mutant was significantly impaired, while its binding to CNX was increased, long-lasting and temperature-independent. The glycan bound to N366 may crucially contribute to the N1234 phenotype, as the phenotype of the N4 mutant (which lacks the N366-linked glycan) shares several features with that of the N1234 mutant. The present findings point to a lectin-independent chaperone activity of CNX on GlyT2, as inferred by its interaction with the N1234 mutant. Co-expression of CNX and N1234 rescued the mutant activity, although not the surface transporter, suggesting that CNX selectively facilitates the surface expression of the more competent conformational state in a glycan-independent manner.

The final degradation of GlyT2 and the N1234 mutant transporter occurs via the lysosomal pathway. The greater increase in the amount of mature transporter observed for the wild type in the presence of lysosomal inhibitors may indicate the existence of more than one degradation pathway for the mutant. However, given the side effects produced by proteasome inhibition, it remains unclear whether the mutant is more prone to proteasomal degradation. In summary, we describe some of the processes involved in GlyT2 biogenesis in the early secretory pathway, including CNX assistance and binding interactions, and we reveal a key role for CNX in the selection of functionally-competent GlyT2 folding intermediates. In the light of this framework, it may now be possible to decipher the effects of hyperekplexia mutations on the plasma membrane expression of newly synthesized GlyT2 transporters.

## Supporting Information

Figure S1 **GlyT2 immunodetection with antibodies against N-terminal and C-terminal epitopes.** COS7 cells expressing wt-GlyT2 or GlyT2 N-terminal (ΔN-GlyT2) or C-terminal (ΔC-GlyT2) deletion mutants were lysed and subjected to Western blot with antibodies against GlyT2 N-terminus (Nt-GlyT2 Ab) or C-terminus (Ct-GlyT2 Ab). ΔC-GlyT2 mutant lacks last 53 amino acids in the GlyT2 C-terminus. ΔN-GlyT2 mutant lacks first 140 N-terminal amino acids of GlyT2 (Poyatos et al., 2000 Molecular and Cellular Neuroscience 15, 99–111). Tubulin immunoreactivity was used as a loading control.
(TIF)

Figure S2 **Time course of expression of N4 mutant.** COS7 cells expressing wtGLYT2 or the N366D (N4) mutant were pulse-labeled for 15 min with [^35^S]methionine/cysteine, chased for the indicated times, immunoprecipitated with GlyT2 antibody and resolved in SDS-PAGE. (A) Kinetics of expression of total synthesized protein. (B) Densitometric analysis of the fluorographies representing labeled bands as a percentage of total synthesized protein.
(TIF)

Figure S3 **Long term lysosomal degradation of GlyT2.** COS7 cells expressing GlyT2 were treated with vehicle or 0.1 mM chloroquine (CQ) during 1 h and then pulse-labeled for 15 min with [^35^S]methionine/cysteine, chased for the indicated times in the absence or presence of the inhibitor, immunoprecipitated with GlyT2 antibody and resolved in SDS-PAGE. Histograms: densitometric analysis of the fluorographies (n = 2–4). Significantly different from the control at the corresponding chase time: *p<0.05 and **p<0.01 in Student's t-test.
(TIF)
