# Supplementary figures and images for: Calnexin-Assisted Biogenesis of the Neuronal Glycine Transporter 2 (GlyT2)
Source: PLoS One. 2013 May 1;8(5):e63230. doi: 10.1371/journal.pone.0063230 (PMC3641136; doi:10.1371/journal.pone.0063230)

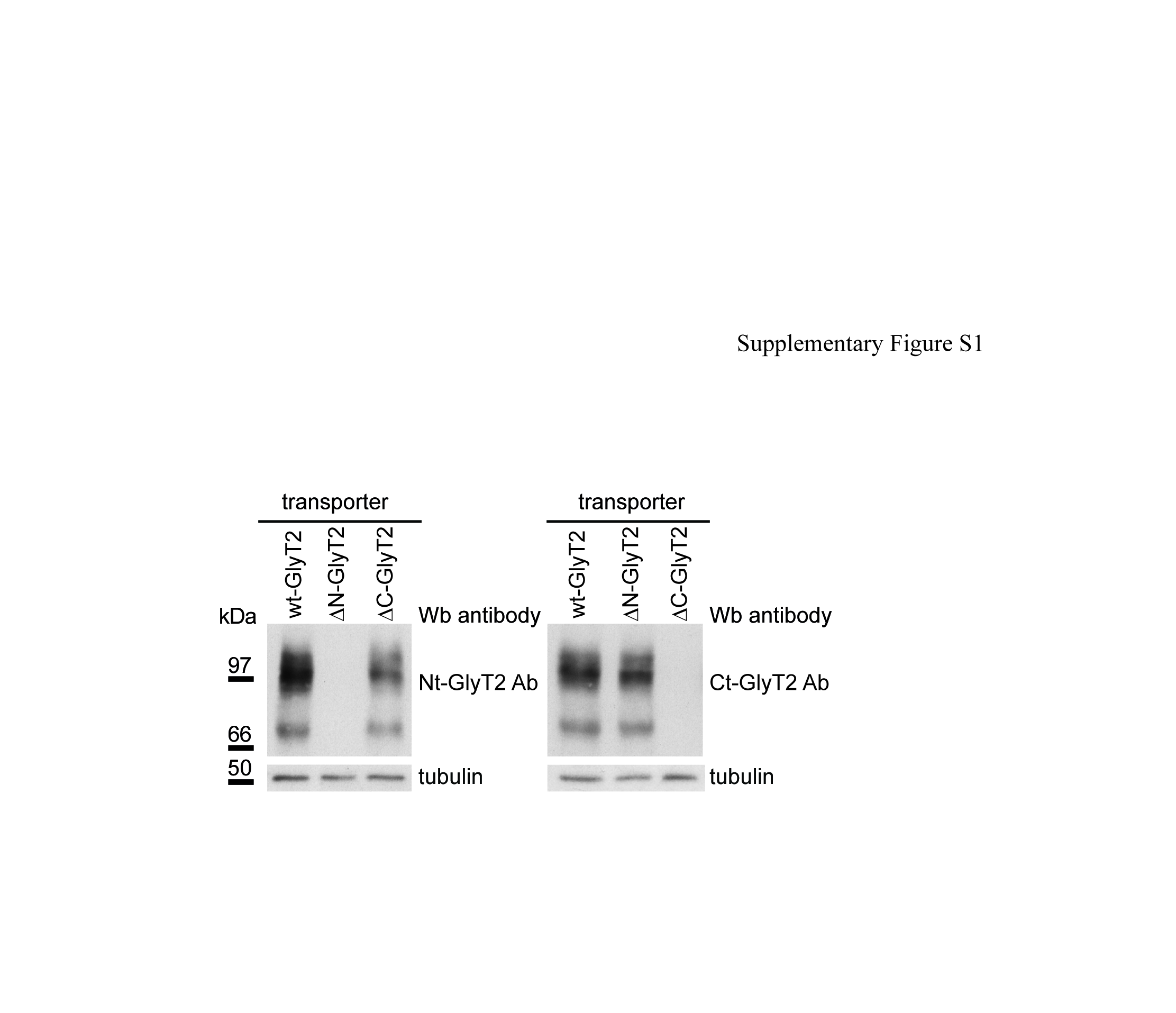

Supplement: Figure S1 — GlyT2 immunodetection with antibodies against N-terminal and C-terminal epitopes. COS7 cells expressing wt-GlyT2 or GlyT2 N-terminal (ΔN-GlyT2) or C-terminal (ΔC-GlyT2) deletion mutants were lysed and subjected to Western blot with antibodies against GlyT2 N-terminus (Nt-GlyT2 Ab) or C-terminus (Ct-GlyT2 Ab). ΔC-GlyT2 mutant lacks last 53 amino acids in the GlyT2 C-terminus. ΔN-GlyT2 mutant lacks first 140 N-terminal amino acids of GlyT2 (Poyatos et al., 2000 Molecular and Cellular Neuroscience 15, 99–111). Tubulin immunoreactivity was used as a loading control. (TIF) [file pone.0063230.s001.tif]

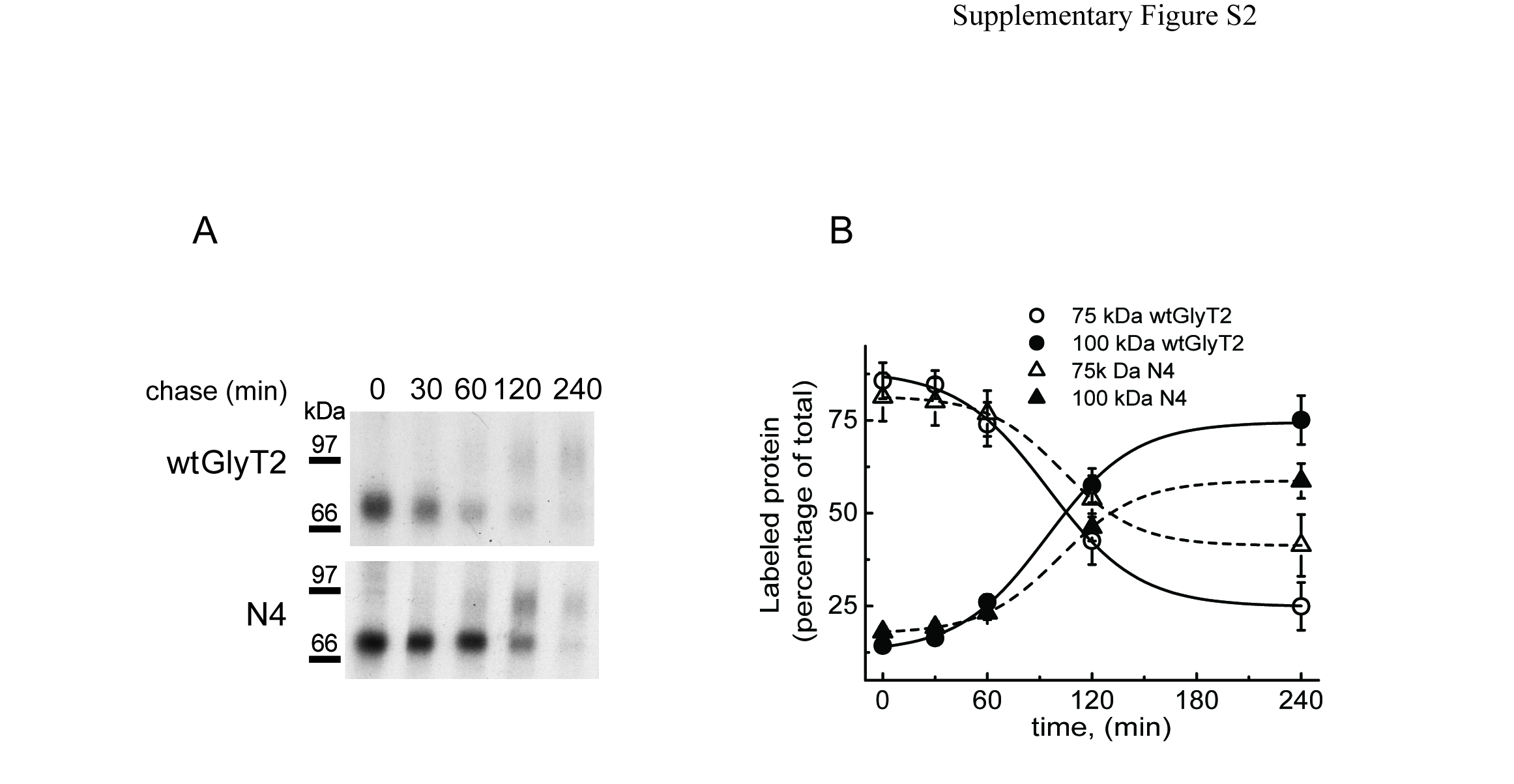

Supplement: Figure S2 — Time course of expression of N4 mutant. COS7 cells expressing wtGLYT2 or the N366D (N4) mutant were pulse-labeled for 15 min with [35S]methionine/cysteine, chased for the indicated times, immunoprecipitated with GlyT2 antibody and resolved in SDS-PAGE. (A) Kinetics of expression of total synthesized protein. (B) Densitometric analysis of the fluorographies representing labeled bands as a percentage of total synthesized protein. (TIF) [file pone.0063230.s002.tif]

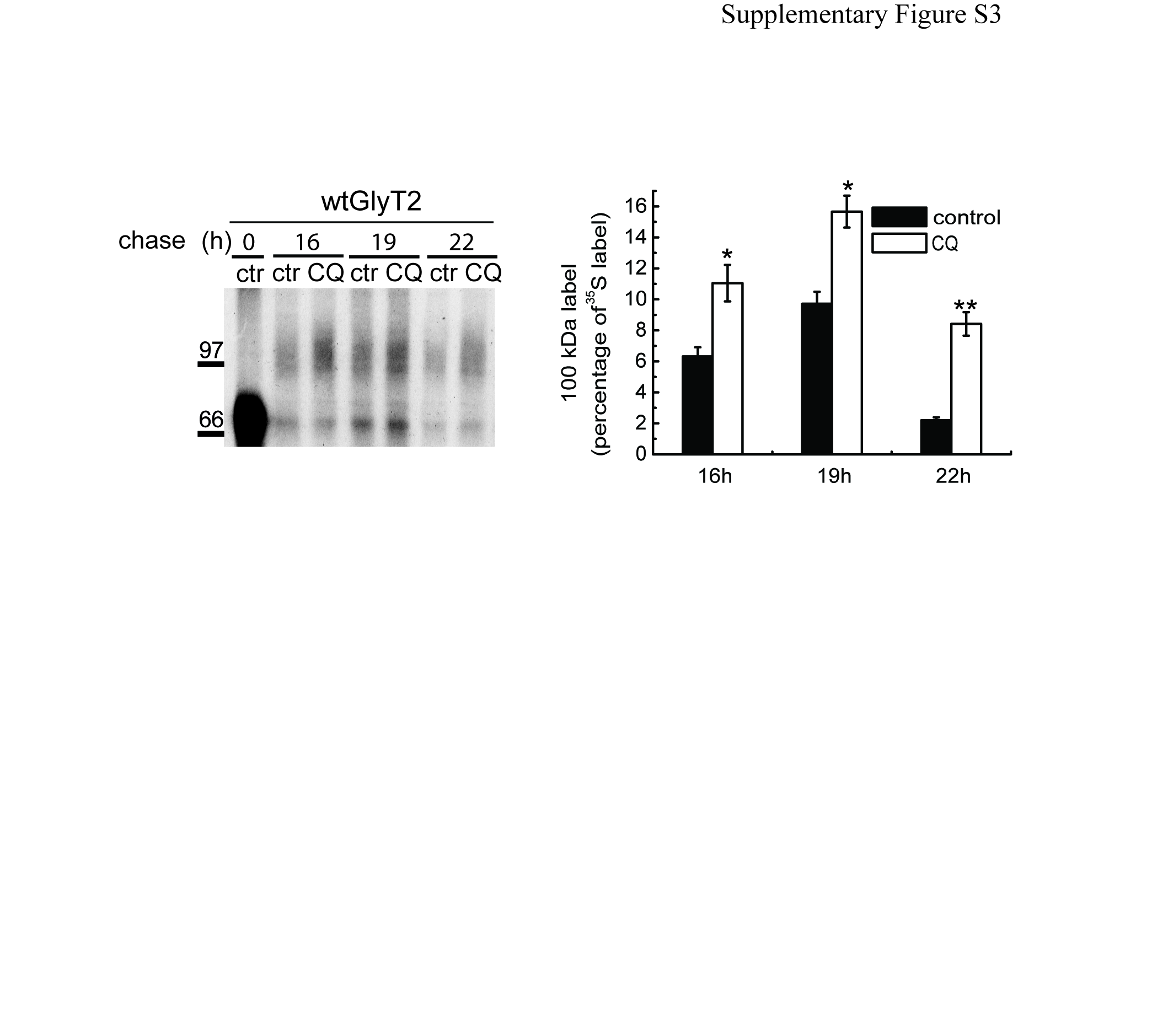

Supplement: Figure S3 — Long term lysosomal degradation of GlyT2. COS7 cells expressing GlyT2 were treated with vehicle or 0.1 mM chloroquine (CQ) during 1 h and then pulse-labeled for 15 min with [35S]methionine/cysteine, chased for the indicated times in the absence or presence of the inhibitor, immunoprecipitated with GlyT2 antibody and resolved in SDS-PAGE. Histograms: densitometric analysis of the fluorographies (n = 2–4). Significantly different from the control at the corresponding chase time: *p<0.05 and **p<0.01 in Student's t-test. (TIF) [file pone.0063230.s003.tif]
